# Supplementary material for: Impact of initial vancomycin pharmacokinetic/pharmacodynamic parameters on the clinical and microbiological outcomes of methicillin-resistant Staphylococcus aureus bacteremia in children
Source: PLoS One. 2021 Apr 1;16(4):e0247714. doi: 10.1371/journal.pone.0247714 (PMC8016336; doi:10.1371/journal.pone.0247714)
Supplement: Attachment — Submitted filename: Response to reviewers_2021 02 10.docx [file pone.0247714.s003.docx]

Reviewer #1: Thank you for addressing my comments. It is a pleasure to review your updated manuscript.

1. Abstract, line 32-33: Rephrase to make clear C trough was measured X times within the time period of Jan. 2010 to March 2018.

- Thank you for the comments. Only 89% (65/73) of the patients measured C_trough_ at least twice. To clarify, I modified the sentence as follows;

C _trough_ was measured at least one time within the time period of January 2010 to March 2018.

2. Abstract, line 36: Make clear that the 47.5% you are referring to is for mechanically ventilated patients (vs ICU admissions). Adding a comma after "intensive care unit stay" should help to clarify that.

- To make it clear, I changed the sentence as follows;

Previously, “Severe cases required intensive care unit stay and mechanical ventilation occurred in 47.5% (35/73)

In this revised one, “Severe clinical outcomes leading to intensive care unit stay and/or use of mechanical ventilation occurred in 47.5% (35/73)

3. Abstract, line 37 and 45: "mortality" used more commonly than "fatality"

=> Thanks for the comments. Throughout the text, we have modified the term fatality by substituting the term mortality.

4. Abstract, line 41, 44, 47: In general, I am noticing "hours" is abbreviated "hrs" or "hr". This should be consistent throughout; I would probably just keep the word spelled out as "hours" in your manuscript text.

- According to the opinion, it was unified and revised in “hours”. Thanks.

5. Introduction, line 51: "major health concerns" is very broad/indirect. One of the major health concerns for what? Pediatrics? Hospitalized patients?

=> Thanks for the comments. To make it clear, it has been modified as follows;

In the previous manuscript, “Methicillin-resistant *Staphylococcus aureus* (MRSA) is one of the major health concerns,”

In the revised one, “Methicillin-resistant *Staphylococcus aureus* (MRSA) is one of the major health concerns in both healthcare-associated and community-acquired infections in children,”

6. Introduction, line 56: cite

- Reference 1 was added after the sentence as follows; Vancomycin is an important treatment option for invasive MRSA infections [1].

7. Introduction, line 58: You say "studies" but only give one citation

=> Reference 3 is a review paper on PK/PD of vancomycin. Thanks.

8. Discussion, page 20, line 4: "good relationship" should be changed to "positive correlation" or something else more objective.

- I changed it to “positive correlation” instead of “good relationship”.

9. Discussion, page 20, line 5: "good linear relationship" - same comment as above

- I changed it to “positive correlation” instead of “good linear relationship”

10. Discussion, page 21, line 10: Please use quotations and define "MIC creep" when first introduced in your manuscript.

- According to your comments, I modified the previous sentences and use as follows;

Even though it is difficult to say clearly that vancomycin “MIC creep” was observed during this study, which was defined as a gradual increase in the central tendency of the vancomycin MIC above 1 or 1.5 μg/mL for the dominant wild-type population [26],

11. Discussion, page 21, line 11: Use >/= consistently vs switching to spelling out "greater than". Also, "MIC creep" is when MIC is > 1, not equal to or greater than 1, and still reported as susceptible.

- Thanks for the points. I modified the sentence as follows;

there was no increased trend of the proportion of MRSA isolates with vancomycin MIC > 1.0 μg/mL.

12. Discussion, page 21, line 23-24: Rephrase; "could expose them to adverse effects such as nephrotoxicity or ototoxicity" or something similar that implies the potential only (and ototoxicity should be mentioned)
=> Thank you for the comments. We changed the previous sentence as follows;

In the previous manuscript, “In addition, targeting vancomycin trough levels > 15 µg/mL in pediatrics would expose them to unnecessary adverse events of nephrotoxicity.”

In the revised one, “In addition, targeting vancomycin trough levels > 15 µg/mL in pediatrics could expose them to adverse effects such as nephrotoxicity or ototoxicity.”

Thank you for addressing my previous comments so thoroughly. I am grateful for the opportunity to review this manuscript again.

- We sincerely appreciate your meticulous review of our manuscript and great expert opinion.

Reviewer #2: I am satisfied that the authors have made revisions according to previous reviewer comments submitted.

- Thank you for reviewing our manuscript and for your informative expert opinion. Thank you again.
